# Specific detection of form IA RubisCO genes in chemoautotrophic bacteria

**DOI:** 10.1002/jobm.201800136

**Published:** 2018-05-24

**Authors:** Albin Allreider, Teresa Bogensperger

**Affiliations:** 1 Institute of Ecology, University of Innsbruck, Innsbruck, Tirol, Austria; 2 Department for Internal Medicine IV, Hospital Wels Grieskirchen GmbH, Wels, Austria

**Keywords:** chemoautotrophic bacteria, CO_2_ fixation, PCR assay, RubisCO

## Abstract

The analysis of RubisCO genes is a highly useful instrument to explore the diversity of chemoautotrophic bacteria using the Calvin–Benson–Bassham cycle for CO_2_ fixation. However, because of the wide taxonomic distribution of phylogenetically related RubisCO forms, environmental studies targeting chemoautotrophs are hampered in habitats dominated by phototrophs. Here, we report the development of a gene marker that specifically detects form IA RubisCO genes in bacteria, excluding photoautotrophic representatives. The high specificity of the PCR assay was confirmed by sequence analysis of DNA obtained from the photic zone of six lakes, were chemoautotrophs are outnumbered by Cyanobacteria also using form IA RubisCO for CO_2_ assimilation.

Ribulose-1,5-bisphosphate carboxylase/oxygenase (RubisCO) is the key enzyme of autotrophic fixation in the Calvin–Benson–Bassham cycle and considered to be the most abundant protein on earth [1,2]. Phylogenetic analyses based on the conserved large subunit genes of RubisCO revealed several forms (I, II, III), which occur in ecologically and evolutionary diverse organisms from all taxonomic domains [3]. Especially form I enzymes show substantial diversification and have been divided into several separate groups. Forms IA and IB belong to the green like group and occurs in *Proteobacteria*, cyanobacteria, green algae, and higher plants. Forms IC and ID are associated with the red like group and are mainly found in *Proteobacteria* and non-green (red) algae [3]. Molecular markers targeting different forms of the large subunit RubisCO gene are a highly specific instrument to explore the diversity of autotrophic organisms using the Calvin–Benson–Bassham cycle for CO_2_ fixation. Several primers pairs were also developed for monitoring chemoautotrophic bacteria, which harbor IA, IC, and II of RubisCO [4–12]. However, because of the extensive evolutionary diversity of organisms using the Calvin–Benson–Bassham cycle, it is not possible to specifically target chemoautotrophs with a single set of molecular markers. Particularly studies focusing on chemoautotrophic bacteria based on genes coding for green like form IA RubisCO are dealing with a major specificity issue in environments populated by high numbers of oxygenic photoautotrophs [6]. To our knowledge, none of the primer sets published so far were designed to exclude photoautotrophic representatives; this includes protocols developed for diversity studies based on RubisCO sequences and quantitative analysis performed with real time PCR. The aim of this study was to develop a gene marker that provides selectivity against nontarget oxygenic phototrophs using RubisCO form IA for CO_2_ fixation, but amplifying a broad spectrum of form *cbbL*-IA genes in bacteria. The efficacy of the PCR-based assay was evaluated with samples from the surface water of six different lakes, where RubisCO form IA sequence libraries have been shown to be dominated by cyanobacterial sequences when amplified with conventional primers sets [6].

For DNA analyses, lake water samples from lakes Achensee (ACH), Egelsee (EGE), Hechtsee (HEC), Piburger See (PIB), Starnberger See (STA), and Zürichsee (ZUR) were concentrated on polyethersulfone filters (pore size 0.22 μm, Millipore, Bedford, USA) and filters were immediately frozen until use. Detailed characteristics of the lakes are given in Alfreider et al. [6]. DNA extraction was performed with the PowerWater® DNA isolation kit (Mo Bio Laboratories Inc., Carlsbad, USA) according to the manufacturer’s protocol. The degenerate forward primer IA_CHEM (5′-GAR GGN TCN GTN GTY AAC GT-3′), specifically selecting against RubisCO form IA in photoautotrophs, was designed based on multiple alignment of form IA sequences from >200 photoautotrophic and chemoautotrophic representatives obtained from GenBank and environmental sequences published by Alfreider et al. [6]. A section of the alignment, including all organisms listed in Table 1, is shown in Supporting Information Fig. S1. For PCR, primer IA_CHEM was combined with form IA reverse RubisCO primer cbbL_IA_r (5′-GTA RTC GTG CAT GAT GAT SGG-3′; Table 1) using a Maxima Hot Start PCR Master Mix (Thermo Scientific Inc., Waltham, USA) following the manufacturer’s instructions. After evaluation of the optimal annealing temperature based on temperature gradient experiments, the following cycle parameters are recommended: 4 min at 95 °C, followed by 35 cycles of 30 s at 95 °C, 30 s at 52 °C, and 1 min at 72 °C, concluding with a 5-min incubation at 72 °C. PCR products were separated on 1.5% agarose gels and bands with proper size were cut out of the gel and purified using the MinElute® Gel Extraction Kit (Qiagen Inc., Valencia, USA). PCR products selected for subsequent cloning were ligated into pGEM-T-Easy Vector plasmid (Promega Corporation, Madison, WI, USA) and transformed into JM109 competent cells following the manufacturer’s instructions. Clones were screened for the presence of proper inserts by PCR using vector-specific primers M13-F/R and GoTaq® G2 Hot Start Master Mix (Promega) following the protocol provided by the manufacturer. Selected reactions were Sanger sequenced by a sequencing service enterprise (Eurofins MWG Operon, Ebersberg, Germany). Closest relatives to deduced amino acid sequences were obtained from public databases (http://www.ncbi.nlm.nih.gov/BLAST/ and http://img.jgi.doe.gov/) and aligned using the MUSCLE algorithm as implemented in MEGA 6.0 software [13]. Neighbor-Joining trees were constructed applying gamma distribution as the distance method in combination with bootstrap analysis (1000 replicates). Sequences data have been submitted to GenBank database under accession numbers MG845266–MG845392.

Table 1 compares the *in silico* coverage of primers developed in this work and primer sets widely used for the detection of form IA(/IB) RubisCO genotypes in environmental studies. The gene marker cbbL_IA_CHEM was designed to encompass a functionally wide range of bacterial chemolithoautotrophs and matches with 70% of proteobacterial representatives (*n* = 115) from taxonomically different phyla (Table 1). On the other hand, primer cbbL_IA_CHEM selects against different phototrophic representatives that contain RubisCO form IA sequences, based on at least two mismatches. In addition to the taxa listed in Table 1, over 200 cyanobacterial sequences retrieved from Genbank were also evaluated *in silico* in terms of selectivity against nontarget sequences. They all showed no cross specificity to the marker cbbL_IA_CHEM (data not shown). All other primers often used for environmental studies (forward and reverse, Table 1) may have significant limitations to detect specifically form IA genes hosted by chemoautotrophs: several of these markers do not cover important taxa and/or recover sequences affiliated with cyanobacterial representatives. Depending on the investigation strategy, primer cbbL_IA_CHEM can be combined with primers listed in Table 1 to produce PCR amplificates with suitable length for different applications (qPCR, next generation sequencing analysis and Sanger sequencing).

To empirically investigate the degree of cross specificity of the newly designed RubisCO form IA marker cbbL_IA_-CHEM to photoautotrophs, DNA extracts from surface water samples of six different lakes were amplified using the newly designed primer cbbL_IA_CHEM in combination with a degenerated reverse primer widely used for environmental investigations (cbbL_IA_r; Table 1). Clone sequences generated from PCR-products were compared with the results of a former study [6], where in the same samples form IA RubisCO genes were amplified based on the same reverse primer primers (cbbL_IA_r) and a forward primer that encompass also photoautotrophs (cbbL_IA_f, Table 1). In this former investigation, most sequences from the same water samples were related to cyanobacterial lineages harboring form IA of RubisCO. In contrast, primer pair cbbL_IA_CHEM/cbbL_IA_r selectively amplify genes of chemoautotrophs over those of photoautrophs and only three of 127 clone sequences were affiliated to *Paulinella chromatophora* lineage that was revovered from the surface water of lakes ACH and STA (Fig. 1). The majority of the sequences (124), however, were related to chemoautotrophs containing form IA RubisCO genes, demonstrating a significant reduction of non-specific PCR-products in samples retrieved from habitats dominated by non-target sequences. Phylogenetic analysis showed that chemo-autotrophic representatives were mostly related with *Betaproteobacteria*, numerically dominated by two distinct clades. One major cluster retrieved from four lakes (ACH, PIB, STA, and ZUR) was affiliated with members of the *Nitrosomonas oligotropha* lineage (cluster 6a), which are considered to be adapted for growth at low substrate concentrations [14,15]. The closest cultivated relative of the second major clade, which was detected in four lakes (ACH, PIB, STA, and ZUR), is the hydrogen-utilizing *Comamonadaceae* bacterium H1 [16]. However, the phylogenetic affiliation of this cluster of RubisCO sequences to different functional traits, including *Thiobacillus* and *Nitrosomonas*, allows only limited interpretation on the ecophysiology and associated energy metabolisms of this group of chemoautotrophs.

In conclusion, our results suggest that the PCR assay developed in this study is effective for preferentially amplifying bacterial RubisCO form IA sequences related to chemoautotrophic organisms in environmental samples. The inclusiveness of target sequences and the exlusion of nontarget photoautotrophic organism by PCR was not only demonstrated based on *in silico* analysis, but also upon the specificity in empirical tests of lake water samples dominated by cyanobacterial RubisCO genes.

## Supplementary Material

Figure S1

## Figures and Tables

**Figure 1:**
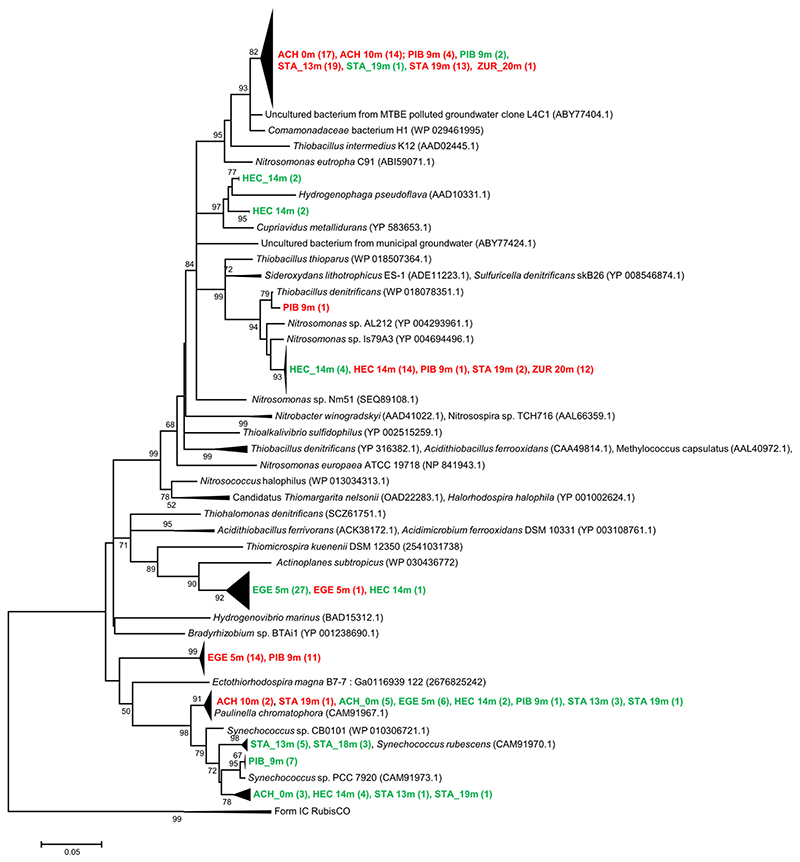
Neighbor-Joining tree from deduced amino acid sequences of form IA RubisCO genes reflecting the coverage of two different primer sets of samples derived from six different lakes. Bootstrap values are shown as percentages of 1000 replicates and values over 50% are indicated on nodes. Scale bar indicates 5% changes in the amino acid sequence. Numbers in brackets show the number of clones. The green color indicates RubisCO form IA lineages that were amplified using primer pair cbbL_IA_f/cbbL_IA_r (Table 1), shown in red are sequences obtained by the newly designed marker gene cbbL_IA_CHEM developed in this study. Representative sequences including closest relatives of cultivated organism were derived from NCBI and IMG databases

**Table 1:** Comparison of the specificity and coverage of different primers against representative photoautotrophic and chemoautotrophic bacteria harboring RubisCO form IA, which were selecet from >200 organisms used for the design of forward primer cbbL_IA_CHEM

Organism	Taxonomic affiliation	Forward primer						Reverse primer					
		cbbL_IA_CHEM^a^ (307-326)	cbbLG11F^b^ (343-362)	RubIgF^c^ (571-590)	cbbL_IA_f^d^ (168-191)	cbbL_f^e^ (571-590)	K2f^f^ (496-15)	cbbL_IA_r^d^ (766-786)	cbbLG1R^b^ (1356-1376)	cbbL1106r^g^ (1106-1129)	RubIgR^c^ (1123-1142)	cbbL_r^e^ (1363-1382)	V2r^f^ (970-990)
Total coverage (%) of 115 proteobacterial representatives tested (no mismatches allowed)		**70**	**31**	**70**	**39**	**3**	**43**	**44**	**17**	**89**	**77**	**10**	**34**
*Acidithiobacillus ferrooxidans* (X7355)	*Acidithiobacillia*												
*Actinoplanes subtropicus* (NZ_JOJL116)	*Actinobacteria*												
*Acidimicrobium ferrooxidans* (CP1631)	*Actinobacteria*												
*Nitrobacter winogradskyi* (AF19915)	*Alphaproteobacteria*												
*Comamonadaceae* bacterium H1 (NZ_BAWN111)	*Betaproteobacteria*												
*Hydrogenophaga pseudoflava* (U5537)	*Betaproteobacteria*												
*Limnohabitans* sp. 63ED37-2 (CP11774)	*Betaproteobacteria*												
*Nitrosomonas halophila* strain Nm1 (FNOY11)	*Betaproteobacteria*												
*Nitrosomonas* sp. AL212 (CP2552)	*Betaproteobacteria*												
*Nitrosospira sp*. TCH716 (AF459718)	*Betaproteobacteria*												
*Sulfuricella denitrificans* skB26 (AP1366)	*Betaproteobacteria*												
*Thiobacillus* sp. SCN 64-317 (MEGO125)	*Betaproteobacteria*												
*Hydrogenovibrio marinus (AB1227)*	*Gammaproteobacteria*												
Oxygenic photoautotrophs													
*Planktothrix agardhii* (CM283)	*Cyanobacteria*												
*Prochlorococcus marinus* (NZ_JNAL115)	*Cyanobacteria*												
*Synechococcus rubescens* (AM71775)	*Cyanobacteria*												
*Synechococcus sp.* CB25 (NZ_ADXM148)	*Cyanobacteria*												
*Synechococcus sp.* PCC792 (AM71776)	*Cyanobacteria*												

Number of mismatches 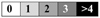 The numbers in brackets below the primer names refer to target nucleotide positions of the primers to the *cbbl* IA gene of *Nitrobacter winogradskyi* strain IFO14297 (AF109915)

^a)^This study; ^b)^Selesi et al. [10]; ^c)^Spiridonova et al. [11]; ^d)^Alfreider et al. [4]; ^e)^Elsaied and Naganuma [7]; ^f)^Nanba et al. [9]; ^g)^Tourova et al. [12].
