# Supplementary material for: Specific detection of form IA RubisCO genes in chemoautotrophic bacteria
Source: J Basic Microbiol. Author manuscript; Available in PMC 2021 May 18. (PMC7610800; doi:10.1002/jobm.201800136)
Supplement: Figure S1 [file EMS124248-supplement-Figure_S1.pdf]

METHOD PAPER

Specific detection of form IA RubisCO genes in chemoautotrophic bacteria

Albin Alfreider[1], Teresa Bogensperger[1,2]

[1]Institute of Ecology, University of Innsbruck, Austria

[2]Department for Internal Medicine IV, Hospital Wels Grieskirchen GmbH, Austria

DOI 10.1002/jobm.201800136

FIGURE S1 Alignment of *cbbL* form IA sequences encompassing the region used for primer design. The annealing position of the primer is underlined and bases are shown in bold. Base mismatches between the primer and the template sequences are indicated by red letters.

Primer cbbL_IA_CHEM	**GARGGNTCNGTNGTYAACGT**
Acidithiobacillus ferrooxidans (X7355)	TACCCCATCGATCTCTTCGAA**GAAGGTTCCGTGGTCAACGT**CTTCACCTCGCTGGTT
Actinoplanes subtropicus (NZ_JOJL116)	TACCCCCTCGATCTCTTCGAG**GAGGGATCCGTGGTCAACGT**CCTGACGTCGCTGGCC
Acidimicrobium ferrooxidans (CP1631)	TACCCGATCGACCTCTTCGAA**GAGGGCTCGGTCGTCAACGT**CTTCACATCGCTGGTC
Nitrobacter winogradskyi (AF19915)	TACCCCATCGACCTTTTTGAG**GAAGGCTCGGTCGTCAACGT**GTTCACCTCGCTGGTG
Comamonadaceae bacterium H1 (NZ_BAWN111)	TACCCGATCGACCTGTTCGAA**GAGGGCTCGGTGGTCAACGT**GTTTACCTCGCTGGTG
Hydrogenophaga pseudoflava (U5537)	TACCCGATCGACCTGTTCGAG**GAGGGCTCGGTGGTCAACGT**ACTGACCTCGCTGGTG
Limnohabitans sp. 63ED37-2 (CP11774)	TACCCCATCGACCTGTTCGAA**GAGGGCTCGGTCGTCAACGT**GCTGACCTCTTTGGTC
Nitrosomonas halophila Nm1 (FNOY11)	TATCCGATCGATTTGTTTGAA**GAAGGTTCGGTGGTCAACGT**GTTCACCTCGTTGGTC
Nitrosomonas sp. AL212 (CP2552)	TACCCGATTGATCTTTTTGAA**GAAGGTTCCGTCGTTAACGT**GTTGACCTCCCTGGTA
Nitrosospira sp. TCH716 (AF459718)	TATCCCATCGACCTTTTTGAA**GAAGGTTCGGTTGTCAACGT**GTTCACCTCGCTGGTG
Sulfuricella denitrificans skB26 (AP1366)	TACCCGATCGACCTGTTCGAA**GAAGGATCCGTCGTCAACGT**TCTGACTTCGCTGGTG
Thiobacillus sp. SCN 64-317 (MEGO125)	TACCCAATCGACCTGTTCGAG**GAAGGCTCGGTCGTCAACGT**GCTGACCTCGCTGGTC
Hydrogenovibrio marinus (AB1227)	TACCCACTAGACCTATTTGAA**GAAGGTTCTGTGGTTAACGT**ATTGACTTCATTGGTT
Oxygenic Photoautotrophs	
Planktothrix agardhii (CM283)	TATCCTCTGGATCTGTTTGAA**GAAGGTTCCGTTACCAACTT**ACTGACATCTTTAGTC
Prochlorococcus marinus (NZ_JNAL115)	TATCCTTTAGATCTTTTTGAA**GAAGGCTCAATTACAAACGT**ATTAACATCATTAGTA
Synechococcus rubescens (AM71775)	TATCCCCTCGACCTGTTCGAA**GAAGGCTCCATCACCAACGT**TCTGACCTCCTTGGTC
Synechococcus sp. CB25 (NZ_ADXM148)	TACCCCCTCGATCTGTTCGAA**GAGGGTTCCGTCACCAACGT**TCTGACCTCCCTGGTC
Synechococcus sp. PCC792 (AM71776	TATCCCCTCGACCTGTTCGAG**GAGGGGTCGATCACCAACGT**GCTCACCTCCCTGGTG
Primer cbbL_IA_CHEM	**GARGGNTCNGTNGTYAACGT**
